# Swine T-Cells and Specific Antibodies Evoked by Peptide Dendrimers Displaying Different FMDV T-Cell Epitopes

**DOI:** 10.3389/fimmu.2020.621537

**Published:** 2021-02-03

**Authors:** Patricia de León, Rodrigo Cañas-Arranz, Sira Defaus, Elisa Torres, Mar Forner, María J. Bustos, Concepción Revilla, Javier Dominguez, David Andreu, Esther Blanco, Francisco Sobrino

**Affiliations:** ^1^Microbes in Health and Welfare Unit, Centro de Biología Molecular “Severo Ochoa” (CSIC-UAM), Madrid, Spain; ^2^Departament de Ciències Experimentals i de la Salut, Universitat Pompeu Fabra, Barcelona, Spain; ^3^Centro de Investigación en Sanidad Animal (CISA-INIA), Madrid, Spain; ^4^Departamento de Biotecnología, Instituto Nacional de Investigación y Tecnología Agraria y Alimentaria (INIA), Madrid, Spain

**Keywords:** foot-and-mouth disease virus, vaccines, dendrimer peptide, T cell phenotype, cytokine

## Abstract

Dendrimeric peptide constructs based on a lysine core that comprises both B- and T-cell epitopes of foot-and-mouth disease virus (FMDV) have proven a successful strategy for the development of FMD vaccines. Specifically, B_2_T dendrimers displaying two copies of the major type O FMDV antigenic B-cell epitope located on the virus capsid [VP1 (140–158)], covalently linked to a heterotypic T-cell epitope from either non-structural protein 3A [3A (21–35)] or 3D [3D (56–70)], named B_2_T-3A and B_2_T-3D, respectively, elicit high levels of neutralizing antibodies (nAbs) and IFN-γ-producing cells in pigs. To assess whether the inclusion and orientation of T-3A and T-3D T-cell epitopes in a single molecule could modulate immunogenicity, dendrimers with T epitopes juxtaposed in both possible orientations, i.e., constructs B_2_TT-3A3D and B_2_TT-3D3A, were made and tested in pigs. Both dendrimers elicited high nAbs titers that broadly neutralized type O FMDVs, although B_2_TT-3D3A did not respond to boosting, and induced lower IgGs titers, in particular IgG2, than B_2_TT-3A3D. Pigs immunized with B_2,_ a control dendrimer displaying two B-cell epitope copies and no T-cell epitope, gave no nABs, confirming T-3A and T-3D as T helper epitopes. The T-3D peptide was found to be an immunodominant, as it produced more IFN-γ expressing cells than T-3A in the *in vitro* recall assay. Besides, in pigs immunized with the different dendrimeric peptides, CD4^+^ T-cells were the major subset contributing to IFN-γ expression upon *in vitro* recall, and depletion of CD4^+^ cells from PBMCs abolished the production of this cytokine. Most CD4^+^IFN-γ^+^ cells showed a memory (CD4^+^2E3^−^) and a multifunctional phenotype, as they expressed both IFN-γ and TNF-α, suggesting that the peptides induced a potent Th1 pro-inflammatory response. Furthermore, not only the presence, but also the orientation of T-cell epitopes influenced the T-cell response, as B_2_TT-3D3A and B_2_ groups had fewer cells expressing both cytokines. These results help understand how B_2_T-type dendrimers triggers T-cell populations, highlighting their potential as next-generation FMD vaccines.

## Introduction

Foot-and-mouth disease (FMD), an acute and systemic vesicular disease that affects cloven-hoofed animals, is caused by FMD virus (FMDV) from the *Aphthovirus* genus within the *Picornaviridae* family (1). FMD is included in the list of notifiable terrestrial and aquatic animal diseases of the World Organization for Animal Health (OIE), as the fatal impact of recurring FMD outbreaks causes huge economic losses in affected countries (2–4). Vaccination remains the most effective method to control FMD (5, 6), with the current OIE-approved vaccine types consisting of chemically inactivated whole viruses emulsified with different adjuvants (7). Although these conventional vaccines have demonstrated their success in eliciting protective immunity against the disease in endemic countries, they have shortcomings such the need for a cold-chain to preserve antigenicity, high-containment biosafety facilities, and difficulties to distinguish infected from vaccinated animals (DIVA capability), among others. These drawbacks underlie non-vaccination policies in some countries (8).

In the face of these limitations, alternative strategies, for instance peptide-based subunit vaccines targeting FMDV protein VP1 have been successfully used to induce anti-FMDV neutralizing antibodies (9). Advantages of such peptide vaccines include: (i) safety, as a non-infectious material is required, and no reversion to virulence is possible; (ii) DIVA condition; (iii) easy handling and storage, with no cold chain needed; (iv) chemical stability, and (v) efficient, affordable large scale production. However, early reports of livestock immunization with linear peptides showed modest levels of protection in livestock, lower than required for use as commercial vaccines (10, 11) and interest in peptide-based vaccines temporarily waned. However, with the advent of so-called multiple antigenic peptides (MAPs) pioneered by Tam (12), an effective approach to increase peptide immunogenicity was demonstrated, and peptide vaccines staged a comeback. In the context of FMD, our own research has focused on dendrimeric constructions, generically termed B_n_T, where several copies of a FMDV B-cell epitope from the G-H loop of VP1 protein in the FMDV capsid (13, 14) are covalently linked through a Lys core matrix to a FMDV T-cell epitope from a non-structural protein (i.e., originally 3A protein, residues 21–35) (15). The selected B-cell epitope shows amino acid variations among different serotypes while the T-cell epitope is highly conserved and therefore can evoke heterologous responses in swine. Interestingly, two doses of a dendrimeric peptide named B_4_T-3A, displaying four copies of a B cell epitope from type C FMDV linked to T-cell epitope 3A (21–35), was able to protect pigs against homologous FMDV challenge (16). Subsequently, a downsized version, i.e. B_2_T-3A, bearing only two copies of a type O FMDV B-cell epitope and being stable in serum for several hours (17), afforded full protection in swine, even upon a single dose (18–20). These protective responses of B_2_T dendrimers are correlated with the induction of high and long-lasting titers of nAbs and the activation of specific lymphocytes providing T-cell help (19, 21). Besides, such T-cell epitopes can also stimulate T-cell subsets leading to the expression of IFN-γ, a cytokine with a relevant role in the antiviral response (22). In another endeavor, a T-cell epitope at the 3D FMDV protein [3D (56-70)], previously shown to be promiscuous and heterotypic in swine (23), displayed as a B_2_T-3D construct, elicited nAbs titers and IFN-γ-producing cells in similar levels to B_2_T-3A (24). As previous results in our groups with linear FMDV peptides had shown that not only the presence but also the orientation of the T-cell epitope influenced the immune response (25), in this work the T-3A and T-3D T-cell epitopes have been juxtaposed in B_2_TT constructs with the two possible orientations, to assess how tandem display and orientation modulate immunogenicity, specifically the T-cell response, a crucial information for improving vaccine design. Also, the requirement of T-cell epitope inclusion in the peptide vaccine was again confirmed, by comparison with ineffective immunizations with construct B_2_, lacking a T-cell epitope.

## Materials and Methods

### Peptides

The B-cell epitope from FMDV O UKG/11/2001, VP1 (residues 140–158), and the T-cell epitopes 3A (residues 21–35) and 3D (residues 56–70) were synthesized by Fmoc-solid phase synthesis. Bivalent branched dendrimers were prepared by conjugation in solution of two B-cell peptides containing an extra C-terminal Cys (free thiol form) to (one or tandem of two) T-cell epitope moieties N-terminally elongated with Lys residues defining a branching point further derivatized by maleimide units (**Table 1**). A Lys-Lys motif, defining a putative cleavage site for cathepsin D, a protease involved in *in vivo* antigen processing for presentation to the MHC class II molecules, was included in the constructs at one (N-terminal of the T-cell epitope in B_2_T, before the branching) or two positions (as above, plus between the T-cell epitope sequences in B_2_TT tandems). The end products were obtained *via* thiol–maleimide ligation at pH 6.0, purified by reverse-phase liquid chromatography and characterized by mass spectrometry (16–18). Final immunogenic peptide dendrimers were water-soluble and stable either lyophilized or in freeze-dried form. Formulation of peptide vaccines was done at the moment of administration.

**Table 1:** Peptide-based vaccine constructs used in this study.

General structure^a^	B_2_T	B_2_TT	B_2_
	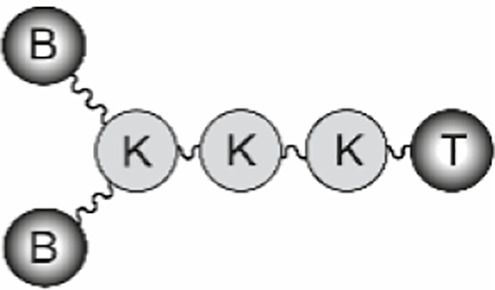	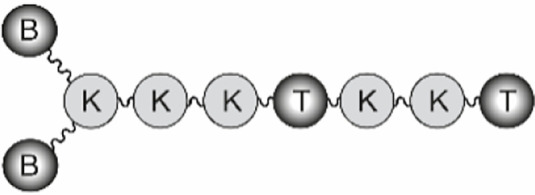	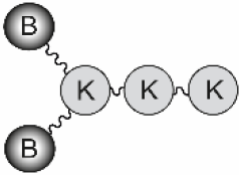
**B**	acetyl-PVTNVRGDLQVLAQKAARTC-amide		
**T-3A**	AAIEFFEGMVHDSIK-amide		
**T-3D**	IFSKHRGDTKMSAED-amide		
**B scrambled**	TGRTVQANVLQDLPAKRA		
**T-3A scrambled**	HMAFESFDGIVKIAE		
**Peptides**	**B_2_T-3A** **B_2_T-3D**	**B_2_TT-3A3D** **B_2_TT-3D3A**	**B_2_**
**MW^b^**	6742.8 Da 6770.8 Da	8708.1 Da	5066.9 Da
**HPLC^c^**	6.9 min (98%) 5.1 min (98%)	6.2 min (98%) 6.0 min (97%)	5.2 min (99%)

^a^Bivalent-branched B-cell epitope immunogens conjugated to none, one or two T-cell epitopes (T-3A or T-3D) in tandem via thiol-maleimide linkages at both α- and ϵ-amino ends of a branched Lys core. ^b^Experimental mass determined by LC/MS. ^c^HPLC retention time (Phenomenex Luna C_18_ column, 4.6×50 mm, 3 μm) eluted with a 20–60% linear gradient of solvent B (0.036% TFA in MeCN) into solvent A (0.045% TFA in H_2_O) over 15 min. ^c^In parenthesis, HPLC homogeneity of purified material.

### Virus

FMDV stocks O UKG/11/2001, O/SKR, O_1_Manisa, O_1_BFS (The Pirbright Institute, UK), and O_1_Campos (OPS-PanAftosa) were amplified in IBRS-2 cells and type C CS8-c1 virus (26) was amplified in BHK-21 cells.

### Animals and Experimental Design

Landrace x Large White female pigs, 9 to 12-week-old (20 Kg), were maintained in a conventional farm facility at the Departamento de Reproducción Animal, INIA, Madrid. Groups of four pigs were immunized with the B_2_T-3A (pigs 49, 54, 65, and 74), B_2_T-3D (pigs 75, 76, 77, 78), B_2_TT-3A3D (pigs 79, 90, 91, and 92) or B_2_TT-3D3A (pigs 93, 94, 95, and 96) constructs at day 0, with 2 ml of Montanide ISA 50V2 emulsion containing 2 mg of the corresponding peptide, and boosted likewise at day 21 pi. In addition, two pigs were immunized and boosted with B_2_ (pigs 97 and 98) and another two PBS-inoculated pigs were maintained as controls (pigs 99 and 100). Because of animal welfare and space requirements, half of the animals (10) corresponding to the lower T-cell responders, were euthanized at day 70 pi. The remaining pigs 10 were housed until the end of the experiment at day 183 pi. Blood samples were collected at days 0, 7, 14, 21, 29, 36, 49, 121, 157, and 183 pi to obtain serum and peripheral blood mononuclear cells (PBMCs). The study was approved (CBS2014/015 and CEEA2014/018) by the INIA Committees on Ethics of Animal Experiments and Biosafety, and by the National Committee on Ethics and Animal Welfare (PROEX 218/14).

### Virus Neutralization Test (VNT)

Neutralization assays were performed in 96-well culture plates. Serial 2-fold dilutions of each serum sample (in DMEM containing 2% fetal bovine serum) were incubated with 100 infection units—50% tissue culture infective doses (TCID_50_)—of FMDV O UKG/11/2001 for 1 h at 37°C. Then, a cell suspension of IBRS-2 cells in DMEM was added and plates were incubated for 72 h. Monolayers were controlled for development of cytopathic effect (cpe), fixed and stained. End-point titers were calculated as the reciprocal of the final serum dilution that neutralized 100 TCID_50_ of homologous FMDV in 50% of the wells (18). For cross neutralization assays, incubation of sera with the panel of FMD viruses (O/SKR, O_1_Manisa, O_1_BFS and O_1_Campos) that belonged to different type O topotypes was performed in parallel to that of the homologous isolate O UKG/11/2001 and the type C CS8-c1 virus as a negative control.

### Detection of Anti-FMDV Antibodies by ELISA

Total anti-FMDV antibodies were determined by means of an indirect ELISA (25). Briefly, 96-well plates coated with peptide B (1µg) were incubated with three-fold dilutions of serum and detected using HRP-conjugated protein A. Plates were read at 450 nm and titers were expressed as the reciprocal of the last serum dilution given an absorbance range of two standard deviations above the background (serum at day 0). Specific IgG1 and IgG2 titers to peptide B were measured and expressed as above using isotype-specific mAbs (Serotec) that were detected with HRP-labeled anti-mouse IgGs (Thermo Fisher). Antibodies against the non-structural protein precursor 3ABC were detected basically as described (18, 27).

### Purification of PBMCs

Porcine PBMCs were isolated from blood samples collected in Vacutainer tubes EDTA-K2, diluted 1:1 in PBS and then used to obtain PBMC by density-gradient centrifugation with Histopaque 1077 (Sigma) and Leucosep tubes (Greiner Bio-One) as described (27). The number of live PMBCs was calculated using a Neubauer chamber (Immune Systems) and cell staining with 0.4% Trypan Blue (Sigma) in PBS. In general, fresh cells were used in the experiments and those remaining were cryo-preserved in 90% FBS and 10% DMSO in liquid nitrogen. The minimum number of cells used for freezing was 2x10^7^/vial.

### IFN-γ Detection by ELISPOT

Quantification of IFN-γ secreting cells from immunized and control animals was performed using an ELISPOT assay. Briefly, 2.5 × 10^5^ PBMCs were shed in triplicate wells of Immobilon-P plates (Merck Millipore) coated as reported (18) and *in vitro* stimulated with 50 µg/ml of their respective dendrimers or with T or B peptides. As positive or negative controls, cells were incubated with 10 µg/ml of phytohaemagglutinin (Sigma) or only with medium, respectively. After 48 h at 37°C and 5% CO_2,_ plates were washed and incubated with a biotinylated mouse anti-pig IFN-γ antibody (clone P2C11, BD) followed by HRP-streptavidin (BD). The frequency of peptide-specific T-cells was expressed as the mean number of spot-forming cells/10^6^ PBMCs, with background values (number of spots in negative control wells) subtracted from the respective counts of stimulated cells. These experiments were performed using outbred domestic pigs with different individual genetic backgrounds. In any case, the levels of animal-to-animal variation did not exceed those observed in other related studies (11).

### Immunoreagents for Flow Cytometry and MACS Cell Sorting

The following mouse anti-pig monoclonal antibodies (mAbs) were used as primary immunoreagents: anti-CD4 (74-12-4, IgG2b) (28), anti-CD8β (PG164A, IgG2a, Eurovet Veterinaria), anti-2E3 (IgM) (29), and anti-CD172a (BL1H7, IgG1) (30). Goat polyclonal Abs (pAbs) specific for mouse Ig subclasses labeled with Alexa647, Alexa488 (Fisher Scientific) or FITC (Bionova) were used as secondary antibodies. For cytokine staining, PE-conjugated anti-pig IFN-γ (P2G10, IgG1) or APC-conjugated anti-human TNF-α (Mab11, IgG1, BD Biosciences) were used. All immunoreagents were titrated for optimal signal/noise ratios. Primary and secondary isotype-matched antibodies were used as negative controls. No background or false staining was observed.

### Intracellular Cytokine Staining (ICS)

To detect the phenotype of cytokine-producing cells, intracellular staining of purified PBMCs was performed using 5x10^6^ PBMCs from each pig that were *in vitro* stimulated or not for 18 h at 37°C with their respective peptide at a final concentration of 25 µg/ml. During the last 10 h, brefeldin A (Sigma) was present in cultures at a final concentration of 5 µg/ml. Medium- or PHA-M-incubated cultures (25 µg/ml) served as negative (non-stimulated) or positive controls, respectively, in a non-immunized animal of each experiment. Cells were washed in PBS-0.05% EDTA-5% FBS and surface stained by incubation with cell-surface primary antibodies for 30 min at 4°C in PBS-0.05% EDTA- 5% swine serum. After three washes in PBS-0.05% EDTA-5% FBS, cells were incubated with the adequate secondary labeled antibodies for 30 min at 4°C. Cells were washed three times, fixed and permeabilized for 20 min at 4°C using Cytofix/Cytoperm buffer (BD). Then, samples were washed in PERM-WASH buffer (BD), free binding sites of secondary antibodies were blocked with whole mouse IgG molecules, and samples were incubated with PE- and APC- conjugated antibodies for the detection of intracellular IFN-γ or TNF-α, respectively, for 30 min at 4°C. Finally, cells were washed three times in PERM-WASH buffer and fixed using a solution containing 2% paraformaldehyde (Electron Microscopy Science). After a further wash, cells were analyzed in a flow cytometer FACS CantoA (BD Biosciences). Data was processed using FACSDiva software (BD Biosciences) or FlowJo software (www.flowjo.com/, Tree Star, Ashland, OR, USA), and transferred to Microsoft Excel for further calculations and preparation of graphs with GraphPad Prism Software 5.0.

### MACS Cell Sorting

For isolation of CD4^+^ T cells, PBMC were incubated with mAb 74-12-4 for 30 min and then washed 2 times with PBS containing 2% FBS and 2.5 mM EDTA and 0.1% sodium azide (MACS buffer). Thereafter, cells were incubated with anti-mouse IgG magnetic microbeads (Miltenyi Biotec, Germany) for 15 min. After a further wash in MACS buffer, the cell suspension was passed through the autoMACS cell sorter and the labeled (CD4^+^) and non-labeled (CD4−) fractions were collected. The purity of each fraction was determined by flow cytometry. Cell staining with anti-CD172a antibody revealed the presence of around 4% of APCs in the CD4^+^ T-cell fraction (**Supplemental Figure 1**). Finally, cells were processed for IFN-γ detection by ELISPOT as described before.

## Results

### Analysis of the Immune Response Induced in Pigs by B_2_T Dendrimers Displaying Two T-Cell Epitopes in Tandem

We first addressed the effect on peptide immunogenicity of including both 3A and 3D T-cell epitopes and their orientation in the same molecule. To this end, T-3A and T-3D T-cell epitopes were combined in a B_2_T-type platform in the two possible orientations, to render peptides B_2_TT-3A3D and B_2_TT-3D3A (**Table 1**). Groups of four pigs were immunized and boosted at day 21 pi, with B_2_T-3A, B_2_T-3D, B_2_TT-3A3D, or B_2_TT-3D3A. Although previous studies with linear peptides had shown that the inclusion of a T-cell epitope was required for inducing optimal immune responses in pigs (31), this requirement for a T-cell epitope in the context of more complex platforms such as our dendrimeric peptides remained to be clarified. Thus, two additional pigs were immunized and boosted with a B_2_ peptide that harbored only two copies of the B-cell epitope without any additional porcine T-cell epitope. Two PBS-immunized animals were included as negative controls.

Since in previous work T-cell responses to B_2_T decreased over time, being more short-lived than antibody responses (24), the frequency of IFN-γ-secreting cells was only analyzed at early times pi (days 7, 14, and 21) and post-boost (days 28 and 35), and by the end of the experiment at day 183 (6 months pi) for those animals that remained alive.

#### Similar Levels of Total IgG Antibodies are Elicited by Dendrimers Harboring One or Two T-Cell Epitopes

**Figure 1:**
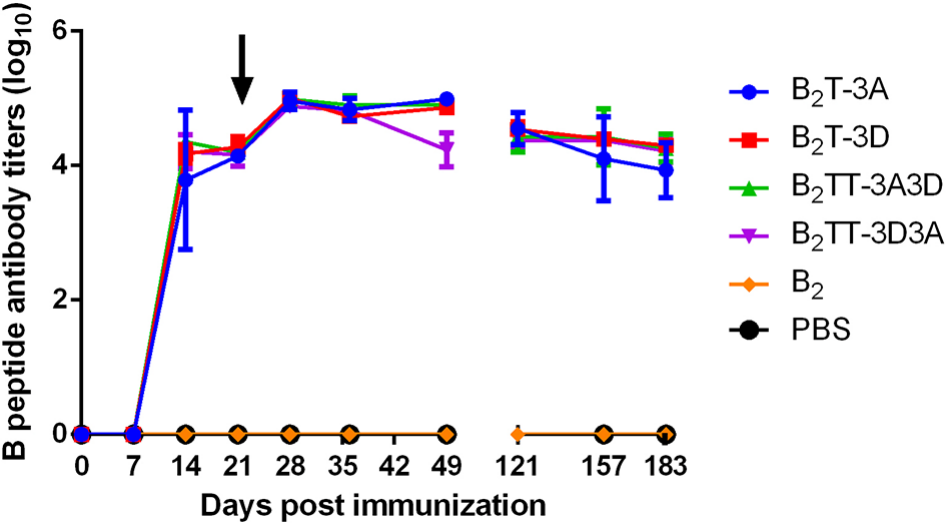
Time course of specific IgG antibodies elicited by B_2_T constructions combining different T-cell epitopes. Each point represents the mean of sera from the indicated group at different days pi (n = 4; except for B_2_ and PBS groups in which n = 2) ± SD. Data on day 121 pi corresponds to two pigs per group. Arrow points the day of peptide boost.

Total IgGs in sera from peptide-immunized pigs were determined by ELISA. All the dendrimer-immunized animals elicited similar levels of specific antibodies, first detected at day 14 pi (about 3.6 log_10_) and slightly increased at day 21 pi (about 4 log_10_). Upon boosting, an increase in IgG titers was observed in the B_2_T-immunized groups at day 28 pi (about 5 log_10_) that remained stable up to day 49 pi, except in pigs immunized with B_2_TT-3D3A, whose titers showed a slight decrease. Interestingly, at day 121 (4 months pi) a sustained antibody response was still observed, with average titers about 4.5 log_10_. This long-lasting antibody response was consistently observed up to the end of the experiment at day 183 (6 months pi) (about 4 log_10_). No antibodies were detected in B_2_ and PBS-immunized groups (**Figure 1**).

#### Neutralizing Activity is Determined by the Presence and Orientation of the T-Cell Epitopes in the Dendrimer

**Figure 2:**
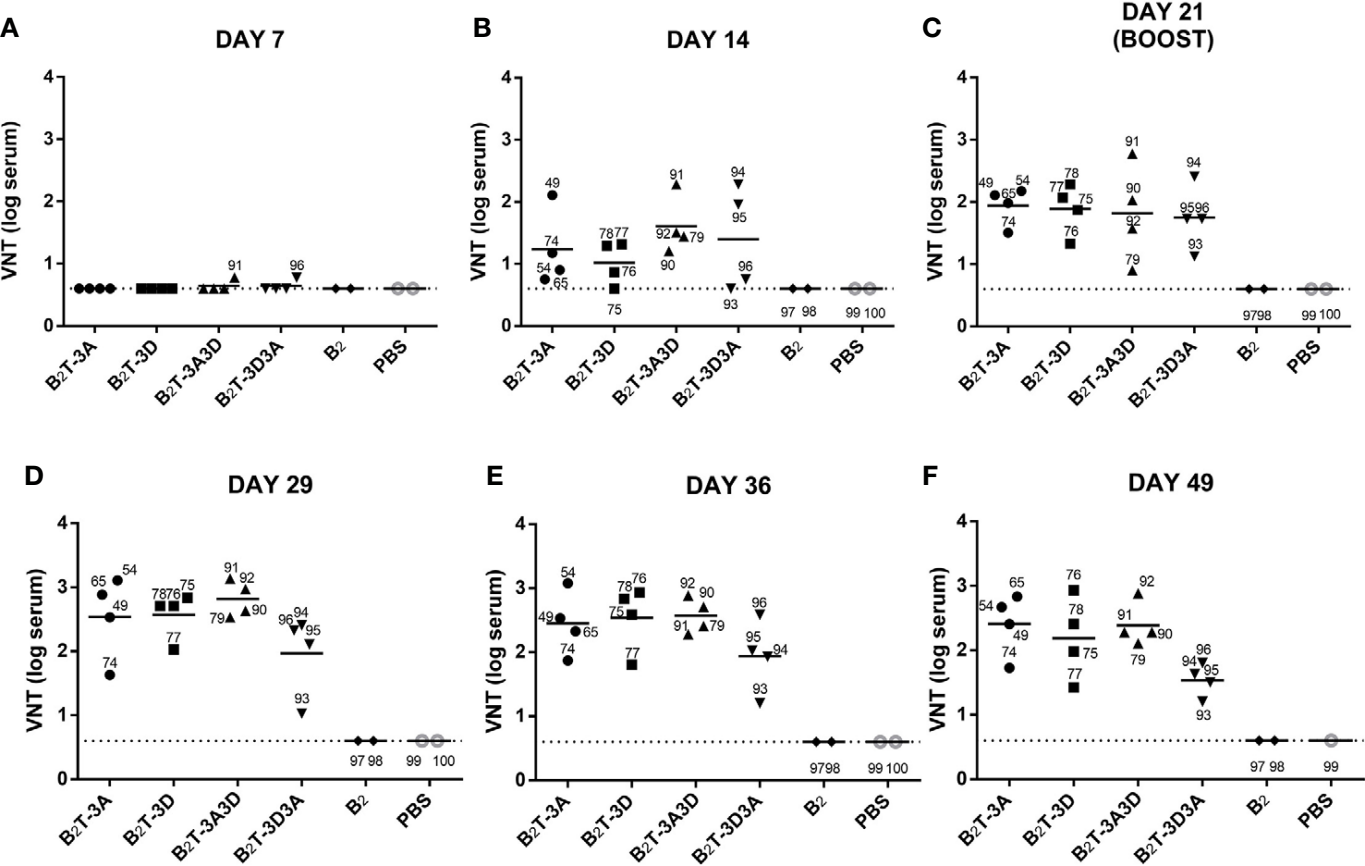
VNTs are influenced by the presence and orientation of the T-cell epitopes in the dendrimer. Sera samples were collected after one dose of each dendrimeric peptide (pre-boost) panels **(A–C)** and after peptide boost [panels **(D–F)**. Each point represents the VNT of one single pig and the mean of each group is shown with a horizontal bar. Dotted line depicts the detection limit.

Neutralizing activity against FMDV was tested in sera from pigs at different times pi (**Figure 2**); nAbs were not detected until day 14 pi (**Figure 2B**). At this time, slightly higher average VNTs were observed for constructions encompassing two T-cell epitopes in tandem (B_2_TT-3A3D: 1.6 ± 0.5; B_2_TT-3D3A: 1.5 ± 0.5 log10) in comparison with those with a single T-cell motif (B_2_T-3A: 1.3 ± 0.6; B_2_T-3D: 1 ± 0.4) (**Figure 2B**). At day 21 pi, an average increment was observed in all dendrimer-immunized groups, reaching similar average titers (B_2_T-3A: 1.9 ± 0.3; B_2_T-3D: 1.9 ± 0.4; B_2_TT-3A3D: 1.6 ± 0.7; B_2_TT-3D3A: 1.7 ± 0.5 log10) (**Figure 2C**). After the boost, at day 28 pi, an increment in VNTs was noticed in pigs immunized with B_2_T-3A (2.5 ± 0.6 log10), B_2_T-3D (2.5 ± 0.4 log10), and B_2_TT-3A3D (2.6 ± 0.3 log10), but not in animals from the B_2_TT-3D3A group (2 ± 0.6 log10) (**Figure 2D**). Titers remained similar over time with minor differences among groups in subsequent days (B_2_T-3A: 2.4 ± 0.5; B_2_T-3D: 2.5 ± 0.5; B_2_TT-3A3D: 2.5 ± 0.3; B_2_TT-3D3A: 1.9 ± 0.5 log10, at day 36 pi; B_2_T-3A: 2.3 ± 0.4; B_2_T-3D: 2.1 ± 0.6; B_2_TT-3A3D: 2.2 ± 0.3; B_2_TT-3D3A: 1.6 ± 0.3 log10, at day 49 pi) (**Figures 2E, F****)**. These differences were not statistically significant.

These results suggested that the presence of T-cell epitopes and their orientation within the dendrimer has an impact in the duration of neutralizing antibody responses against the FMDV G-H loop (B-cell epitope). Indeed, animals that were not euthanized—pigs 49 and 65 (B_2_T-3A), 76, 77 and 78 (B_2_T-3D), 91 and 92 (B_2_TT-3A3D), 93 and 94 (B_2_TT-3D3A)—showed at days 121, 157 and 183 pi (4, 5, and 6 months pi, respectively) VNTs similar to those observed at day 49 pi (data not shown). This result is consistent with previous observations on the long-lasting neutralizing responses elicited by B_2_T-dendrimers (19).

Remarkably, no nAbs were detected at any time point in pigs immunized with the B_2_ peptide, with the branched B-cell epitopes but lacking a T-cell motif. This lack of response confirms the functional role of T-3A and T-3D as T helper epitopes, i.e., inclusion of at least one of them is essential for nAbs to be elicited in pigs.

#### Dendrimers Elicit nAbs Against a Broad Spectrum of Type O FMDVs

**Figure 3:**
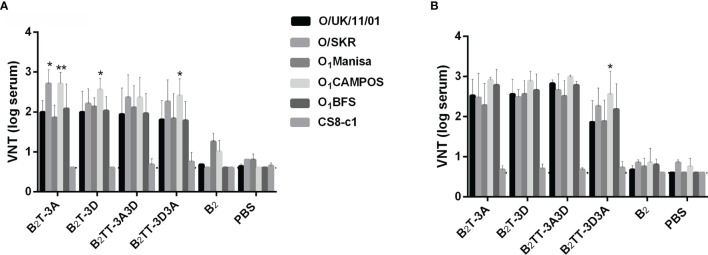
Sera from pigs immunized with dendrimers can neutralize a panel of different FMDVs type O topotypes. Sera recovered from animals immunized with the different peptides after the first (day 21 pi or pre-boost) **(A)** and the second immunization (day 28 pi or post-boost) **(B)**, were tested to neutralize a panel of different type O FMDVs. Individual columns represent the mean of each group (n = 4) ± SD. Values are expressed as the reciprocal log_10_ of the last serum dilution that neutralized 100 TCID50 of each FMDV. Statistically significant differences are indicated by one (*) or two (**) asterisks for *p < 0.05* or *p < 0.005*, respectively.

Since type O FMDVs are responsible for many current FMD outbreaks in endemic countries, a broad-spectrum response is necessary for effective vaccines against this serotype (32). Therefore, we were interested in assessing the neutralization range afforded by B_2_T-dendrimers. Recently, we observed that sera from pigs immunized with B_2_T-3A or B_2_T-3D were able to neutralize a panel of type O FMDVs belonging to various spatiotemporal locations (24). We decided to extend the study to animals vaccinated with B_2_T-dendrimers harboring two different T-cell epitopes in tandem, as well as with B_2_, to assess again the relevance of T-cell epitopes in the induction of neutralizing activity. As shown in **Figure 3**, both constructions with two T-cell epitopes induced nAbs against the panel of FMDVs, showing similar titers before boosting (**Figure 3A**), while only B_2_TT-3A3D had a VNT increase afterwards (**Figure 3B**). Neutralizing activity against viruses such as O_1_Campos and O/SKR appeared to be significantly higher, in some cases, than that against the homologous virus O UKG/11/2001; factors inherent to the assay, such as differential thermal stability among viral isolates, might contribute to explain these observations. As expected, no nAbs were detected in B_2_-immunized animals, confirming our previous results with homologous O UKG/11/2001 virus, nor in all animals tested against isolate CS8-c1 that belongs to a different serotype (**Figure 3**). Thus, B_2_TT-3A3D and B_2_TT-3D3A behave just like previously tested B_2_T-3A and B_2_T-3D, being able to induce a broad anti-FMDV immunity within a serotype.

#### Isotype-Specific IgG1 and IgG2 Antibodies Elicited by Dendrimers B_2_TT-3A3D and B_2_TT-3D3A

**Figure 4:**
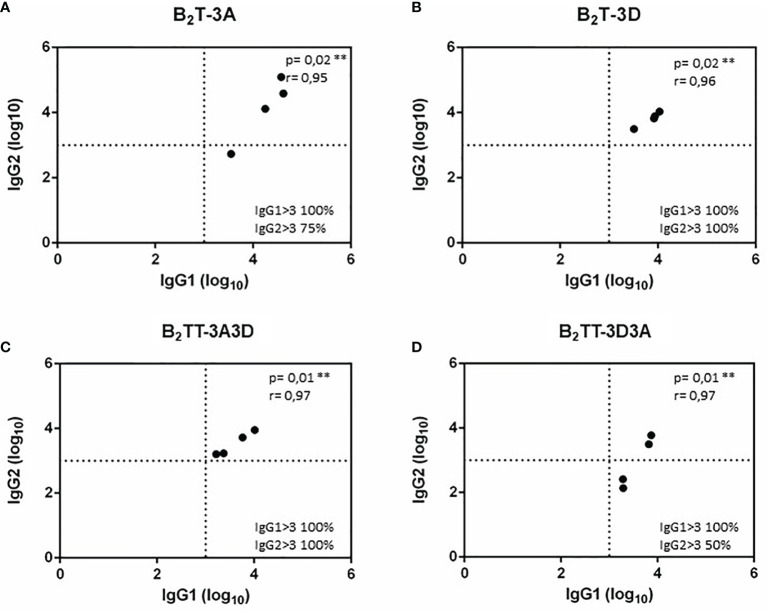
IgG1 and IgG2 profiles elicited by B_2_T constructions combining different T-cell epitopes. Anti-B peptide IgG1 and IgG2 profiles in sera from pigs collected at 21 days pi with: B_2_T-3A **(A)**, B_2_T-3D **(B)**, B_2_TT-3A3D **(C)**, and B_2_TT-3D3A **(D)**. Each point represents the value of a duplicate of an individual animal. Endpoint titers are expressed as the reciprocal of serum dilution (log_10_) giving the absorbance recorded in control wells (sera collected at day 0) ^+^ 2 x SD. Each symbol represents the IgG1 and IgG2 titers (X and Y values respectively) for an individual pig. In the lower right quadrant, the percentage of pigs with titers above 3 log_10_ is shown. No individual spontaneous reactivity was observed in the titers determined at day 0.

To test whether the inclusion of two consecutive T-cell epitopes in the dendrimer and their orientation could favor expression of a specific IgG isotype and influence the class switching, the anti B-peptide IgG1 and IgG2 profile was analyzed by ELISA in serum samples from pigs immunized with the different constructs at day 21 pi. All dendrimers induced specific IgG1 and IgG2 albeit to different extents (**Figure 4**). Three animals from the B_2_T-3A group showed high titers of both IgG1 and IgG2 (about 5 log10) and only one pig did not develop high IgG2 titers (< 3 log_10_) (**Figure 4A**). In the same day, all animals immunized with B_2_T-3D reached similar IgG1 and IgG2 titers (about 4 log10) that were, in average, lower than those of B_2_T-3A group (**Figure 4B**). Moreover, pigs immunized with B_2_TT-3A3D reached isotype titers similar to those of the B_2_T-3D group suggesting that T-3D was modulating the isotype switching (**Figure 4C**). On the other hand, the construction B_2_TT-3D3A induced low (titers < 3 log10) IgG2 titers, in two of the four animals (**Figure 4D**).

#### T-Cell Responses Elicited by B_2_TT-3A3D and B_2_TT-3D3A: Immunodominance of the 3D T-Cell Epitope

**Figure 5:**
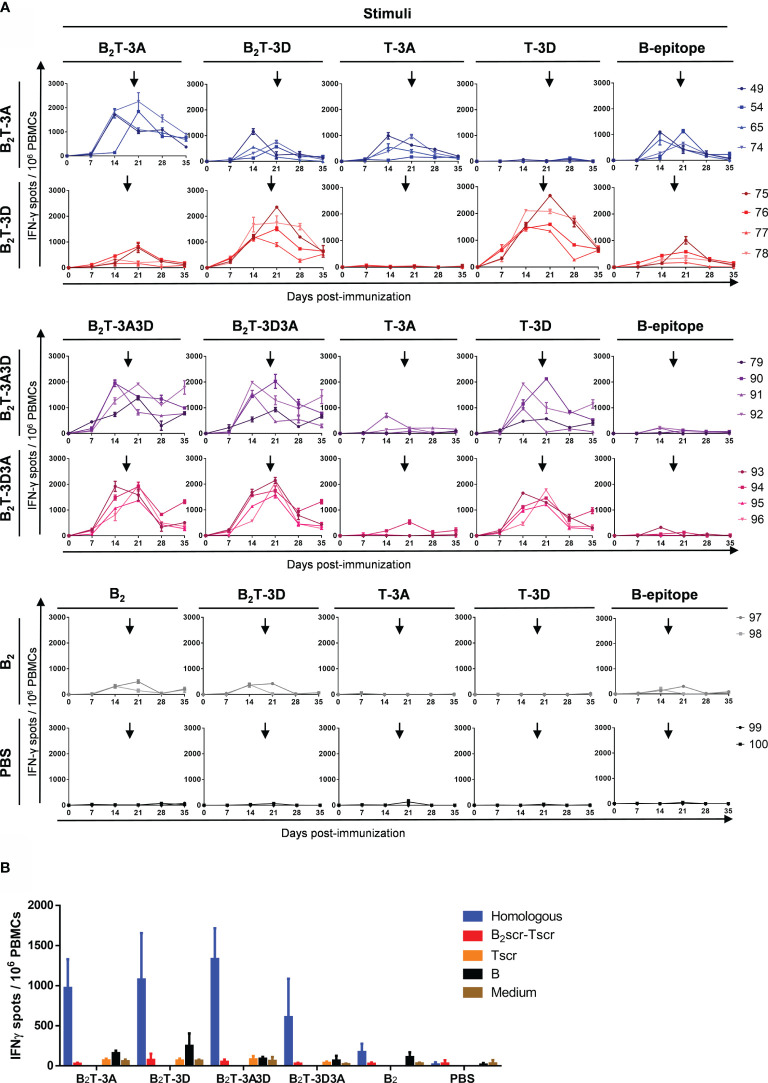
T-cell responses elicited by B_2_TT-3A3D and B_2_TT-3D3A unveil 3D peptide as an immunodominant T-cell epitope. PBMCs were isolated from individual animals of each group, at different days pi. Cells were *in vitro* stimulated for 48 h with dendrimers and the frequency of IFN-γ producing cells was determined by ELISPOT. **(A)** Frequency of IFN-γ secreting cells in the groups of pigs immunized as indicated, in response to the homologous dendrimer and the different constructions indicated. Each line corresponds to an individual animal and arrows show the day of the boost. **(B)** IFN-γ production was stimuli-specific and sequence-dependent. The values corresponded to PBMC recovered at day 183 and are presented as IFN-γ spots per 10^6^ cells). Each column represents the mean (n = 4) ± SD.

The T-cell responses elicited by dendrimers B_2_TT-3A3D, B_2_TT-3D3A, B_2_T-3A, B_2_T-3D, and B_2_ were analyzed using PBMCs collected at different days pi. Cells were *in vitro* stimulated with the homologous peptide as well as with the heterologous one (B_2_TT-3A3D/B_2_TT-3D3A or B_2_T-3A/B_2_T-3D) and with the B- and the 3A and 3D T-cell peptides, in an attempt to further understand the antigenic specificity of the elicited responses (**Figure 5**).

When T-3D and T-3A were joined together in dendrimers B_2_TT-3A3D and B_2_TT-3D3A, a consistent T-cell response was observed in pigs immunized with these constructions. Similar high levels of IFN-γ producing cells were found upon *in vitro* stimulation with the homologous or the heterologous dendrimeric peptides that only differed in the T-cell epitope orientation. Interestingly, the response was higher when cells were *in vitro* stimulated with peptide T-3D than with peptide T-3A, suggesting an immunodominance of T-3D T-cell epitope over T-3A. Low levels of IFN-γ secreting cells were noticed when the B-cell peptide was used as stimulus, unlike what observed in the responses induced in pigs from the B2T-3A and B_2_T-3D groups (**Figure 5A**).

Significant frequencies of IFN-γ expressing cells were detected in the B_2_T-3A group upon *in vitro* recall with the homologous dendrimer. In general, responses were high in all the animals. The number of IFN-γ secreting cells detected was stimulus-dependent, the highest values being induced by the homologous dendrimer B_2_T-3A. Responses to T-3A were also significant, confirming that this peptide was recognized as a T-cell epitope. The specificity of the T-cell response was confirmed as no IFN-γ spots were detected upon stimulation with the heterologous T-cell epitope T-3D. However, a heterologous response against B_2_T-3D peptide was also noticed, similar to that elicited against B-cell epitope. These results point to a contribution of the B-cell epitope present in the B_2_T dendrimers to the effector cellular responses (**Figure 5A**), although we cannot rule out that the overall configuration of the dendrimer platform itself may also play a role in T-cell activation.

Peptide B_2_T-3D was also able to induce similar frequencies of IFN-γ expressing cells in response to peptide T-3D in all the animals. This result confirms that T-3D can be considered a T-cell epitope able to induce an IFN-γ effector immune response. As observed in animals immunized with B_2_T-3A, the B-cell peptide was also able to induce IFN-γ secretion, suggesting, again, that this peptide may be recognized as a T-cell epitope (**Figure 5A**).

Low levels of IFN-γ expressing cells were also detected in animals immunized with peptide B_2._ This poor recognition of B_2_ as a T-cell epitope highlights the decisive contribution of 3A and 3D epitopes to the cellular immune response elicited by the dendrimer peptides. As expected, non-immunized pigs from the control group did not show specific T-cell responses against any of the peptides (**Figure 5A**).

In order to rule out whether the induction of T-cell responses could be influenced by non-specific mechanisms, a dendrimer with a scrambled amino acid sequence (src) in both the B- and the T-3A epitopes (B_2_scr-Tscr) was used to stimulate PBMCs from day 183. No IFN-γ secreting cells were detected neither in response to the whole scrambled dendrimeric peptide (B_2_scr- Tscr) nor to the scrambled T-cell peptide alone (Tscr) (**Figure 5B**), showing that a branched architecture is not enough to induce an effector T-cell response, and that specific T-cell epitope sequences are required.

### Characterization of T-Cell Populations Elicited Upon Immunization With B_2_T Dendrimers

Flow cytometry assays, including both surface marker staining and intracellular cytokine staining (ICS), were performed in dendrimer-stimulated PBMCs from immunized pigs at day 21 pi, in order to evaluate the specific T-cell populations induced upon immunization with B_2_T peptides. To make the assay feasible, the two best responders from each group, in terms of levels of IFN-γ producing cells determined by ELISPOT assays, were selected: pigs 49 and 65 (B_2_T-3A), pigs 76 and 77 (B_2_T-3D), pigs 91 and 92 (B_2_TT-3A3D), pigs 93 and 94 (B_2_TT-3D3A), pig 98 (B_2_) and pig 100 (PBS).

#### ICS Analysis Unveils CD4^+^ T-Cells as the Major Population Involved in T-Cell Response

**Figure 6:**
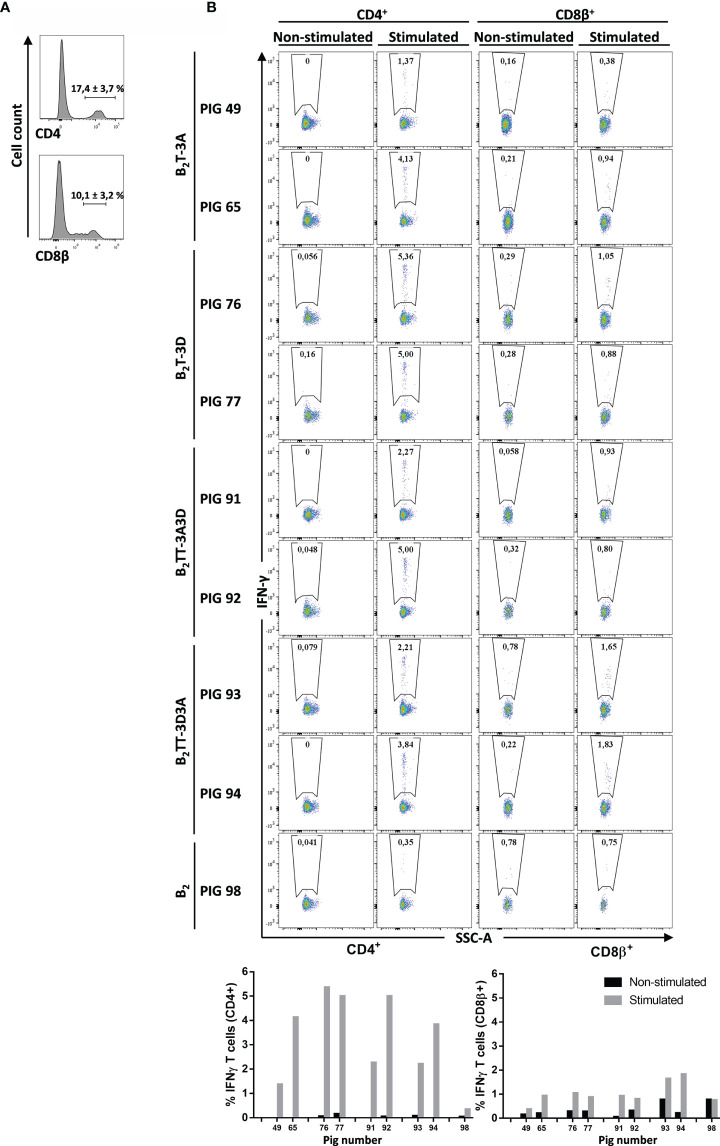
CD4^+^ and CD8β^+^ specific T-cell responses elicited by different dendrimeric peptides in PBMCs from immunized animals. Intracellular cytokine staining was performed in overnight dendrimer stimulated PBMCs from immunized animals at day 21 pi. **(A)** PBMCs were stained and analyzed for surface CD4 or CD8β expression (representative plot from pig #49 is shown). **(B)** CD4^+^ or CD8β^+^ populations from each pig were gated and further analyzed for IFN-γ expression. IFN-γ-producing cells are shown as percent of total CD4^+^ or CD8β^+^ T cells.

PMBCs were stained for CD4 or CD8β expression, followed by intracellular cytokine staining for IFN-γ in CD4^+^ (helper) or CD8β^+^ (cytotoxic) T-cells. The percentages of CD4^+^ and CD8β^+^ populations differed among pigs (17.4 ± 3.7% and 10.1 ± 3.2%, respectively) (**Figure 6A**). Upon specific stimulation, IFN-γ was preferentially expressed by CD4^+^ T-cells in all immunized animals (n = 2). The percentage of CD4^+^IFN-γ^+^ cells was variable, being higher in B_2_T-3D group (5.2 ± 0.3%), followed by B_2_TT-3A3D (3.6 ± 1.9%), B_2_TT-3D3A (3 ± 1.1%), and B_2_T-3A (2.8 ± 2%) groups. Notably, the B_2_-immunized animal (pig 98) hardly induced CD4^+^IFN-γ^+^ T-cells (0.4%), further supporting the need for a T-cell epitope to induce a cellular response, as observed by ELISPOT (**Figure 5**). IFN-γ production was less evident in CD8β^+^ T-cells, where the highest response was found in the B_2_TT-3D3A group (1.7 ± 0.1%), followed by B_2_T-3D (1 ± 0.1%), B_2_TT-3A3D (0.9 ± 0.1), B_2_ [pig 98 (0.8%)] and B_2_T-3A (0.7 ± 0.4%) groups. These results point to CD4^+^ T-cells as the main population involved in dendrimer-induced T-cell immunity (**Figure 6B**).

#### Depletion of CD4^+^ T-Cells from PBMCs Abolishes IFN-γ Production

**Figure 7:**
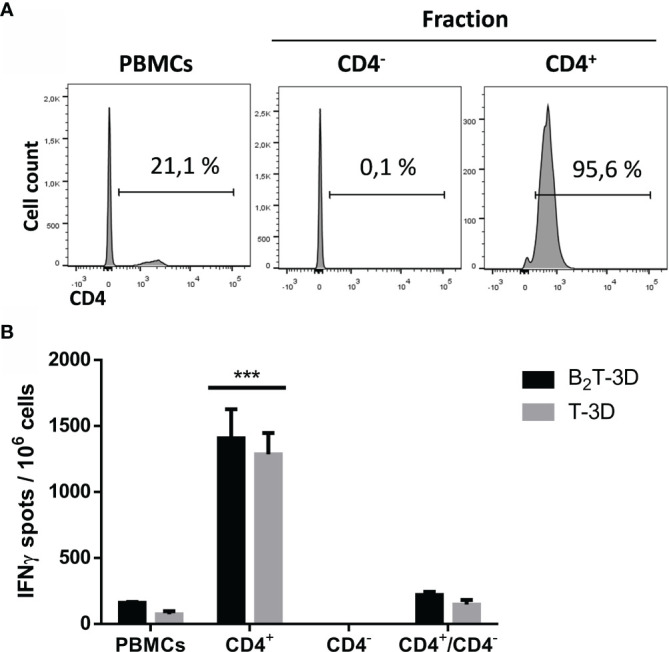
Depletion of CD4^+^ T-cells abolishes IFN-γ production of peptide-stimulated PBMCs. Cells from a B_2_T-3D immunized pig (pig #77) were magnetically sorted using an anti-CD4 mAb. **(A)** FACS analysis was performed to determine the CD4 staining quality of initial pre-sorted PBMCs and each purified fraction after MACS fractioning. **(B)** Pre-sorted PBMCs and fractioned positive fraction (CD4^+^), negative fraction (CD4^−^) and reconstituted fraction (CD4^+^/CD4^−^) were plated in triplicates and stimulated either with B_2_T-3D or T-3D peptides for 48 h and IFN-γ production was determined by ELISPOT. The values are presented as IFN-γ spots per 10^6^ cells (spots from mock-stimulated cells are subtracted). Each column represents the mean of a triplicate ± SD. Statistically significant differences between the positive fraction (CD4^+^) and the rest of the groups are indicated by three asterisks (***) for *p < 0.001*.

To confirm the phenotype of the T-cell subset responsible for IFN-γ expression, CD4^+^ T-cells were depleted of PBMCs isolated from a B_2_T-3D immunized pig (pig #77), which was selected for being a high IFN-γ producer when tested in ELISPOT and ICS assays (**Figures 5** and **6**). The magnetically isolated positive (CD4^+^) and negative (CD4^−^) fractions were *in vitro* stimulated with B_2_T-3D or T-3D peptides and IFN-γ expression was determined by ELISPOT. Unfractioned PBMCs and PBMCs reconstituted with the CD4^+^ and CD4^-^ fractions (CD4^+^/CD4^−^) were included as controls. The efficiency of the isolation process was verified by flow cytometry. As shown in **Figure 7**, 21.1% of initial PBMCs were CD4^+^. After the sorting, a drastic reduction in the number of CD4^+^ cells (0.1%) was found in the eluted negative fraction (CD4^−^) while the positive fraction (CD4^+^) showed a notable enrichment in CD4^+^ (95.6%) (**Figure 7A**). No fluorescence was detected in cells incubated with secondary antibodies alone (data not shown). Both unsorted PBMCs and magnetically sorted fractions were stimulated with B_2_T-3D and T-3D peptides to quantify IFN-γ expression by ELISPOT. As shown in **Figure 7B** only the CD4^+^ cells produce IFN-γ upon stimulation with either the dendrimer or the T cell epitope, while no IFN-γ spots were detected in the CD4^−^ fraction. As expected, total PBMCs and the reconstituted fraction CD4^+^/CD4^−^ showed similar levels of IFN-γ secreting cells. These results confirm that CD4^+^ T-cells are the main population responsible for specific IFN-γ release.

#### Memory CD4^+^ T-Cells Are the Responsible Subset of the IFN-γ Secretion

**Figure 8:**
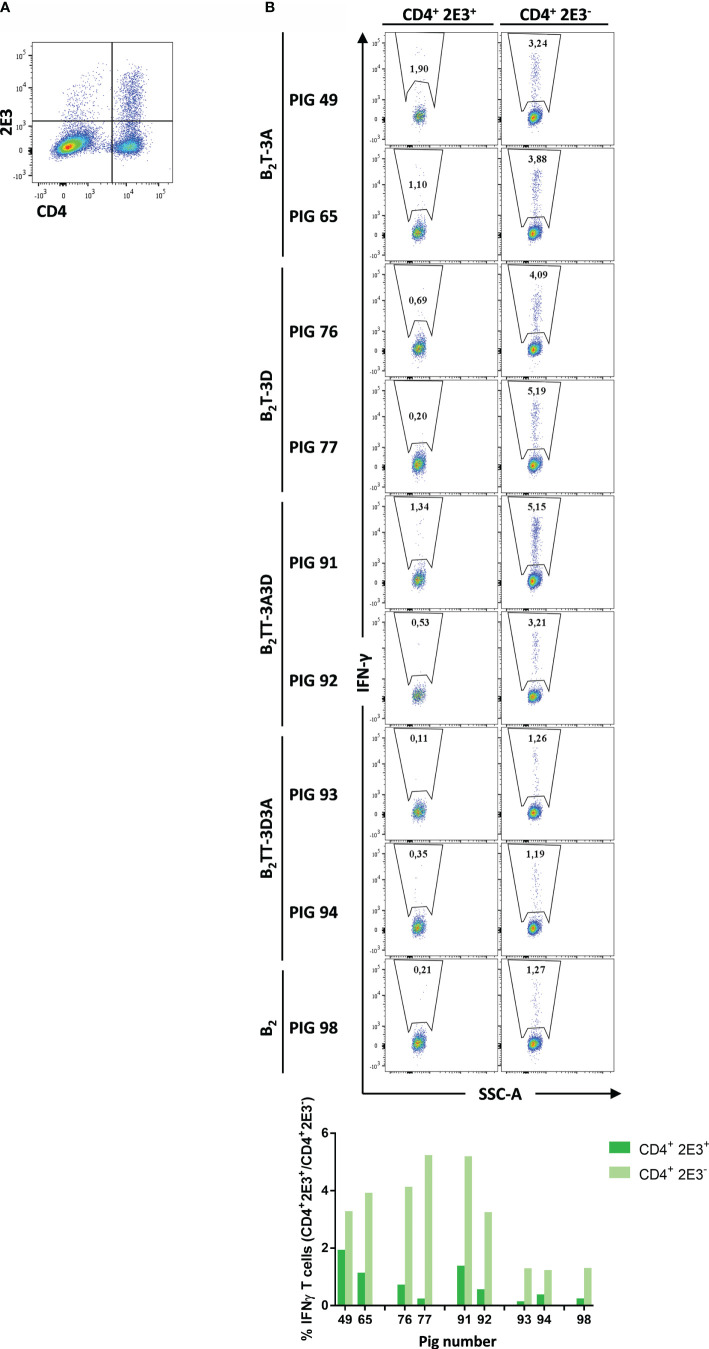
CD4^+^2E3^-^ T-cells are the major subset producing IFN-γ. Intracellular cytokine staining was performed in overnight dendrimer stimulated-PBMCs isolated from immunized pigs at day 14 pi. **(A)** PBMCs were stained and analyzed for surface CD4 and 2E3 expression (representative plot from pig #49 is shown). **(B)** CD4^+^ 2E3^+^ and CD4^+^2E3^−^ populations were gated and further analyzed for IFN-γ expression. IFN-γ-producing cells are shown as percent of total CD4^+^2E3^+^ or CD4^+^2E3^−^ T cells.

2E3 expression has been used in swine immunological studies to differentiate naïve and memory CD4^+^ T-cell subpopulations. This marker is expressed on the surface of naïve CD4^+^ cells in swine PBMCs but not in memory CD4^+^ cells (29, 33). Dendrimer-stimulated PBMCs from immunized pigs were stained for the expression of CD4 in combination with 2E3, and ICS for IFN-γ was performed in both gated CD4^+^ T cell subpopulations (i.e., CD4^+^2E3^+^ and CD4^+^2E3^−^). The percentage of CD4^+^2E3^+^ and CD4^+^2E3^−^ cells in the pigs varied, being higher for the latter subset (3.3 ± 1.3% and 8.9 ± 3.2%, respectively) (**Figure 8A**). CD4^+^2E3^+^ subset showed a low percentage of IFN-γ-expressing cells, with higher levels in the B_2_T-3A group (1.5 ± 0.6%), followed by B_2_TT-3A3D (0.9 ± 0.6%), B_2_T-3D (0.4 ± 0.3%) and B_2_TT-3D3A and B_2_ (0.2 ± 0.2%) groups. A clear induction of IFN-γ was observed in the CD4^+^2E3^−^ subset from all immunized groups, which was higher in B_2_T-3D (4.6 ± 0.8%), B_2_TT-3A3D (4.2 ± 1.4%), and B_2_T-3A (3.6 ± 0.5%) groups than in B_2_TT-3D3A (1.2 ± 0%) and B_2_ (1.3%) groups (**Figure 8B**). No fluorescence signal was detected in non-stimulated cells (data not shown).

These results suggest that memory CD4^+^ T-cells are the major subset involved in IFN-γ production and in the T-cell response elicited by B_2_T dendrimers.

#### Multifunctional CD4^+^ T-Cells Are Activated by the B_2_T Dendrimers

**Figure 9:**
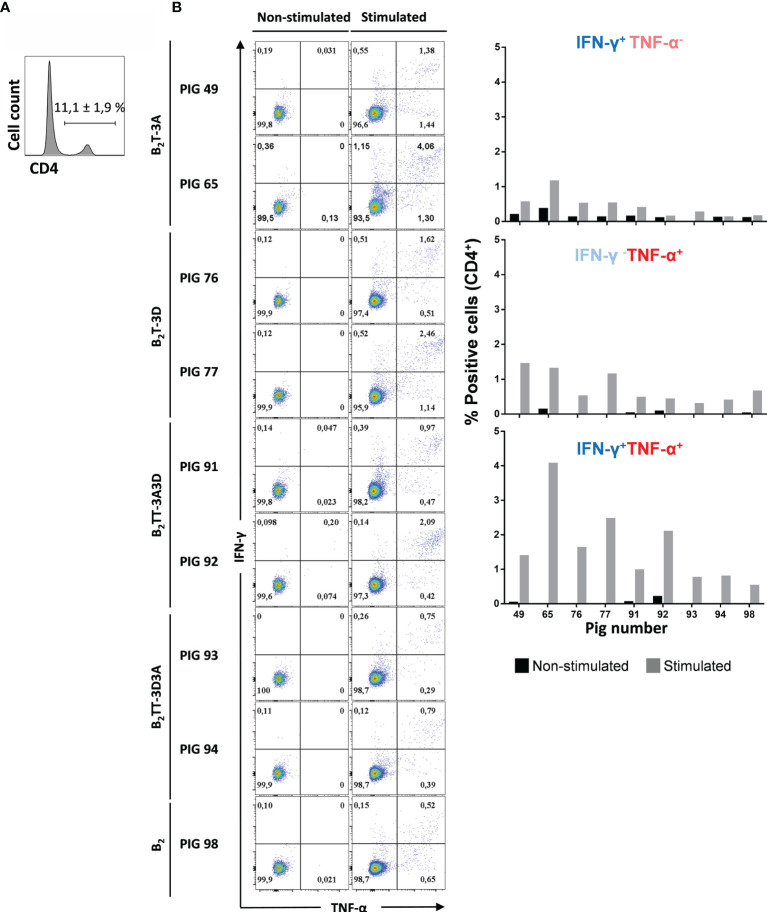
Multifunctionality of CD4 T cells from immunized pigs upon stimulation with homologous dendrimer. Intracellular cytokine staining of PBMCs was performed following overnight *in vitro* stimulation with homologous dendrimers. **(A)** CD4 staining and expression was analyzed (representative plot of animal #49 is shown). **(B)** CD4^+^ T cells were gated and analyzed for production of a single cytokine (IFN-γ^−^TNF-α^+^ and IFN-γ^+^TNF-α^−^ subsets, in upper left and bottom right quadrants) or of both cytokines (IFN-γ^+^TNF-α^+^ subset in upper right quadrant). Cytokine-producing cells are shown as percent of total CD4^+^ T cells.

Studies on multifunctional T-cells, i.e., T-cells performing several functions like cytokine production or degranulation, have revealed their central role in protective immune responses (34). IFN-γ is the main effector cytokine expressed in Th1 T-cells, but other pro-inflammatory cytokines are also involved in Th1 effector responses and are important in T-cell immunity. Therefore, in order to evaluate whether CD4^+^ T-cells activated by the B_2_T dendrimers were producing effector cytokines other than IFN-γ, ICS was performed for IFN-γ and TNF-α expression (**Figure 9A**; a representative plot from pig #91 is shown). When the expression of IFN-γ and TNF-α was analyzed in the gated CD4^+^ T-cell population, a strong fluorescence signal was detected in PBMCs stimulated with homologous peptides (**Figure 9B**).

Most of the activated CD4^+^ T-cells expressed both cytokines, which is consistent with a multifunctional CD4^+^IFN-γ^+^TNF-α^+^ phenotype. This was particularly evident in pigs immunized with B_2_T-3A (2.7 ± 1.9%), B_2_T-3D (2 ± 0.6%), and B_2_TT-3A3D (1.5 ± 0.8%). In contrast, in B_2_TT-3D3A (0.8 ± 0.1%) and B_2_ [pig 98 (0.5%)] groups showed lower levels of cells expressing both cytokines (**Figure 9B**). A lower percentage of CD4^+^ T cells producing a single cytokine CD4^+^IFN-γ^+^TNF-α^−^ and CD4^+^ IFN-γ^−^TNF-α^+^ was also detected with average values of 0.4 and 0.7%, respectively.

In summary, these data indicate that a considerable proportion of dendrimer-responding CD4^+^ T cells is multifunctional in terms of IFN-γ and TNF-α production, and that not only the presence, but also the orientation of T-cell epitopes influences the T-cell response evoked by these peptides.

## Discussion

The protective responses elicited by B_2_T-3A and other related dendrimeric constructs are associated with the induction of high titers of nAb and lymphocytes activation providing a T-cell helper response (16, 18). Such T-helper epitopes can also stimulate T-cell subsets expressing IFN-γ, a cytokine relevant to antiviral response (22). Thus, characterization of the functional role of the T-cell epitope(s) recognized by swine lymphocytes in our B_2_T-type molecules is relevant for optimizing our vaccine constructions. MHC restriction can pose a bottleneck in subunit vaccines development, as T-cell epitope recognition may vary within species with MHC repertoires (35). For this reason, expanding T-cell epitope diversity in dendrimeric vaccines may improve recognition and presentation to genetically different individuals.

Replacement of T-3A or its combination with other T-cell peptides was a reasonable approach to explore alternative recognition of B_2_T-type constructions by T- cells. We previously demonstrated that a B_2_T dendrimer with a T-3D epitope elicits high levels of neutralizing antibodies and a potent IFN-γ response (24). In this work, we have confirmed that B_2_T-3D and B_2_T-3A induce similar nAb titers in pigs, and shown that when juxtaposed in the same molecule, T-3A/T-3D orientation influences the response, as B_2_TT-3A3D, unlike B_2_TT-3D3A, elicited optimal nAb titers, especially after boost. These results evidence differences in the immunomodulation of the antibody response. When compared with the response elicited by a commercial FMD vaccine, the magnitude of the neutralization titers elicited by the dendrimer constructs studied is about 1 log_10_ lower than that evoked by type O (O Campos) commercial vaccine (27), and similar to those elicited by B_2_TA that result in protection against virus challenge (19). Experiments are being arranged to confirm such correlation in the new constructions analyzed. In addition, the failure of a B_2_ construct lacking the T-cell motif to induce antibodies is consistent with the need of specific T-cell help (36). Nevertheless, the influence of other factors such as possible non-functional B-cell epitope conformations in B_2_ resulting in impaired presentation to B- and/or T-cells, cannot be excluded. Interestingly, B_2_T-3A also induced IFN-γ expressing T-cells that were recalled with similar time courses by T-3A and, to a lesser extent, by B peptide suggesting that both sequences were recognized as T-cell epitopes. Conversely, the IFN-γ expressing cells elicited by B_2_T-3D preferentially recognized the T-3D peptide, supporting its dominance. When the two epitopes were combined in a single molecule, B_2_TT-3A3D and B_2_TT-3D3A induced a clear activation of specific T-cells. In fact, when peptides with each of the T-cell epitopes (T-3A and T-3D) were used as stimuli, a marked hierarchy of T-cell responses were observed, peptide T-3D being the main inducer of IFN-γ. The mechanisms underlying the immunodominance of T-3D peptide over the other epitopes (T-3A and B) remain to be clarified.

Regarding the isotype profile of the antibody response, B_2_TT-3A3D induced high IgG1 and IgG2 titers similar to those of the animals immunized with B_2_T-3D and B_2_T-3A, suggesting the ability of T-cell peptides T-3A and T-3D to induce the IgG class-switching, as observed in IFN-γ inductions. Thus, when individually included in a B_2_T-dendrimer, T-3A and T-3D can provide similar T-cell help for the production of antibodies, including nAbs. However, B_2_TT-3D3A induced, in two out of four tested animals, lower titers of both IgG isotypes than B_2_TT-3A3D, indicating that the T-cell epitopes orientation in the dendrimer is important for the IgG class-switching. This difference in immunomodulation is also observed in the lack of boost effect in the nAbs levels induced by B_2_TT-3D3A.

In humans and mice, IFN-γ is mainly produced by T lymphocytes and NK cells (37, 38). The subject is less clear in the case of pigs, reflecting the complexity of the cell subsets involved and the limited availability of specific reagents for the characterization of swine antigens (39, 40). Thus, a wide variety of IFN-γ producing cells are known to be induced by specific stimuli or cytokines, including Th-cells, Tc-cells, NK cells and γδ T-cells, the latter being highly abundant in swine (39, 41, 42). Flow cytometry analyses of PBMCs from peptide-immunized pigs showed a percentage of CD4^+^ and CD8β^+^ positive populations that agreed with those described for outbred 3–5 month-old pig populations (43). Indeed, a strong IFN-γ production was noticed in the CD4^+^ subset derived from vaccinated animals upon homologous stimulation, ranging from 3–5%, depending on the peptide. Although a limited number of animals was used for the analysis (n = 2), B_2_T-3D induced the highest IFN-γ secretion (**Figure 6**). These results correlated with those of the IFN-γ ELISPOT and point to the epitope T-3D as a potent IFN-γ inductor as well as mainly recognized by CD4^+^ cells. In contrast, a limited activation of the cytolytic CD8β^+^ subset was noticed in these experiments (ranging from <1–1.5% depending on the peptide), suggesting a minor contribution of CD8β^+^ cells in the response to the dendrimers. These results are consistent with the kind of T-cell epitopes included in the dendrimers, as they were previously identified as swine T helper epitopes (15, 23). The possibility of eliciting heterotypic CD8 responses against highly conserved FMDV epitopes can interestingly contribute to potentiate the protective responses against this highly antigenic variable virus. Induction of CD8 responses has been described upon vaccination of pigs (44, 45) and cattle (46, 47). Therefore, further understanding of the role that CD8 responses can play in the protection conferred by the B_2_T peptides as well as the development of strategies aimed at increasing activation of CD8 T-cells, are among the goals for improving dendrimeric peptide vaccines.

The importance of CD4^+^ T-cells upon FMDV vaccination is supported by the observation that depletion of this subset *in vivo* ablated antigen-dependent lymphoproliferative responses (48). On the other hand, a CD4^+^ T-cell-independent nAb response has been reported using *in vivo* depletion of this cell subset in a different species (36). The major role of the CD4^+^ cell subset on the response to dendrimer B_2_T-3D was confirmed when this population was depleted from PBMCs. The sorted CD4^-^ subset failed to produce IFN-γ whereas the CD4^+^ enriched fraction substantially produced IFN-γ upon dendrimer and T-peptide stimulation (**Figure 7**).

A fraction of primed T-cells can persist in the absence of specific antigen (memory T-cells) being necessary for a rapid response upon antigen recall (38, 49, 50). Considering that the PBMCs used for the enrichment studies were collected at 3.5 months pi, the presence in them of a substantial proportion of memory T-cells was expected. Indeed, the mild expression of IFN-γ in the CD4^+^2E3^+^ (naïve T cells) in contrast with the strong cytokine detection in the effector/memory CD4^+^2E3^-^ subset, suggests that effector/memory T-cells were responsible of responses in the two animals analyzed from each peptide group (**Figure 8**). This effect was more prominent for B_2_T-3D immunized animals and correlated with ELISPOT results (**Figure 5**), further supporting the capacity of T-3D in eliciting potent Th1 responses.

Not only IFN-γ but other pro-inflammatory cytokines such as TNF-α can drive T-cells towards a Th1 pathway, the main effector cells for viral clearance. Indeed, T cells producing several cytokines, also called multifunctional T cells, are considered to be functionally superior to their single-producing counterparts, and have been associated/correlated with protection in several virus infections (51, 52). Our results showed that in dendrimer-immunized animals the majority of IFN-γ producing CD4^+^ T cells also produced TNF-α, although there were also minor subsets of CD4^+^ IFN-γ^+^TNF-α^-^ and IFN-γ-TNF-α^+^ single producers (**Figure 9**). Interestingly, IFN-γ^+^ and/or TNF-α^+^ producing CD4^+^T-cells were less frequently observed in the B_2_TT-3D3A immunized pigs, as in the B_2_-immunized group/animals, indicating again that the T-cell epitope orientation in dendrimer B_2_TT-3D3A is less efficient in T-cell activation. To our knowledge, this is the first study that addresses in detail the T-cell subsets stimulated upon FMDV immunization with either the whole virus or its subunits. Furthermore, B_2_ peptide was unable to induce IFN-γ in CD4^+^ subsets, strengthening the importance of the incorporation of a specific T-cell epitope for the dendrimers to elicit IFN-γ secreting T cells.

Altogether, our results point to Th1 responses as the main effector mechanism of the T-cell activation elicited by the dendrimeric constructs. The overall results presented support the use of the dendrimer platforms, such as those studied here, as potential FMDV heterotypic next-generation vaccines.

## Data Availability Statement

The original contributions presented in the study are included in the article/**Supplementary Material**. Further inquiries can be directed to the corresponding authors.

## Ethics Statement

The animal study was reviewed and approved by CSIC Committees on Ethical and Animal Welfare (CBS2014/015 and CEEA2014/018) by the INIA Committees on Ethics of Animal Experiments and Biosafety, and by the National Committee on Ethics and Animal Welfare (PROEX 218/14).

## Author Contributions

FS, EB, DA, PL, RC-A, and SD conceived and designed the experiments. PL, RC-A, ET, SD, MF, MB, and CR performed the experiments. PL, RC-A, JD, EB, DA, and FS analyzed the data and wrote the manuscript. All authors contributed to the article and approved the submitted version.

## Funding

The research was supported by the Spanish Ministry of Science, Innovation and Universities grants AGL2014-48923-C2 and AGL2017-89097-C2-2-R (to FS and DA) and AGL2016-349 76445-R to EB), as well as by Comunidad de Madrid co-financed with ECFEDER funds (S2013/ABI-350 2906-PLATESA and P2018/BAA-4370 to FS and EB) and by Generalitat de Catalunya (2009SGR492 to DA). Work at Centro de Biología Molecular “Severo Ochoa” and at UPF was supported by Fundación Ramón Areces and by the Maria de Maeztu Program of the Spanish Ministry of Science, Innovation and Universities, respectively. RC-A and MF were holders of a PhD fellowship from the Spanish Ministry of Science, Innovation and University (FPI programme).

## Conflict of Interest

The authors declare that the research was conducted in the absence of any commercial or financial relationships that could be construed as a potential conflict of interest.
