# Supplementary material for: Swine T-Cells and Specific Antibodies Evoked by Peptide Dendrimers Displaying Different FMDV T-Cell Epitopes
Source: Front Immunol. 2021 Feb 3;11:621537. doi: 10.3389/fimmu.2020.621537 (PMC7886804; doi:10.3389/fimmu.2020.621537)
Supplement: Supplementary file 1 [file Image_1.pdf]

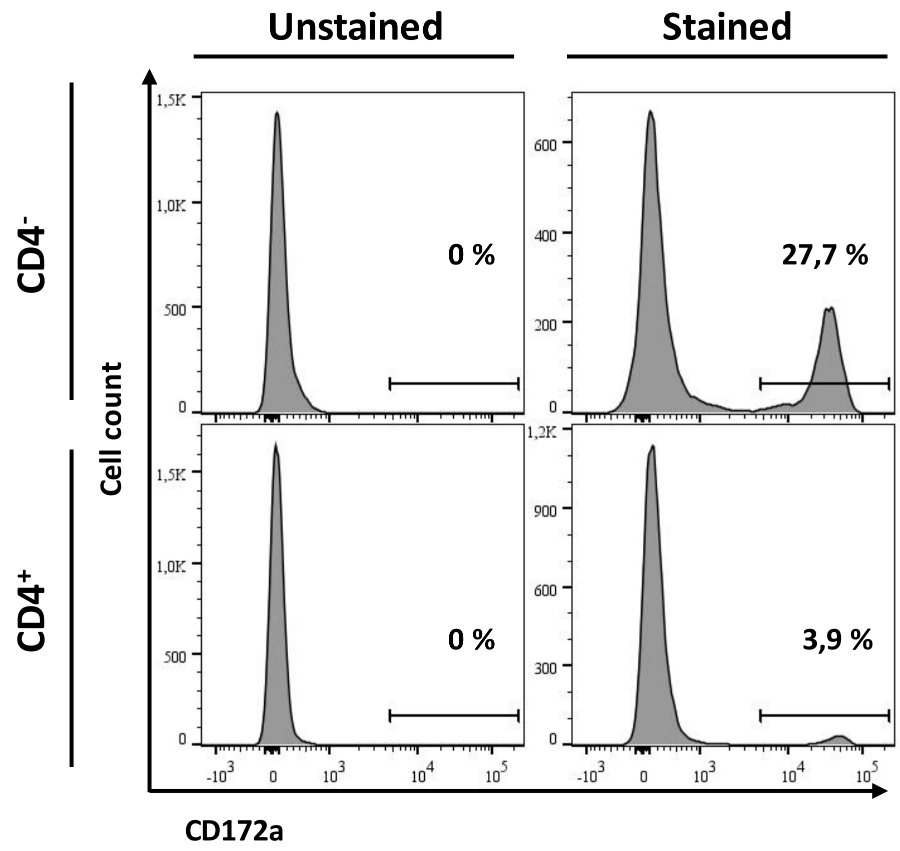

Supplementary Figure 1. The CD4+ fraction contains APCs: Flow cytometry analysis was performed to determine the expression of CD172a, a marker of porcine APCs, in CD4+ or CD4- fraction from fractioned PBMCs.
